# Eating Disorder Symptoms and Energy Deficiency Awareness in Adolescent Artistic Gymnasts: Evidence of a Knowledge Gap

**DOI:** 10.3390/nu17101699

**Published:** 2025-05-16

**Authors:** Anastasia Donti, Maria I. Maraki, Maria Psychountaki, Olyvia Donti

**Affiliations:** School of Physical Education & Sport Science, National & Kapodistrian University of Athens, 17237 Athens, Greece; adonti@phed.uoa.gr (A.D.); mmaraki@phed.uoa.gr (M.I.M.); mpsychou@phed.uoa.gr (M.P.)

**Keywords:** energy deficit, eating behaviors, adolescence, gymnastics, energy availability, aesthetic sports

## Abstract

**Background/Objectives**: Pressure to stay lean may lead adolescent athletes to dietary restraint and disordered eating. Lack of nutrition awareness can also contribute to suboptimal dietary habits, increasing the risk of eating disorders and Relative Energy Deficiency in Sport [RED-S], though evidence in competitive athletes is limited. This study explored eating disorder symptoms and RED-S knowledge in adolescent artistic gymnasts. **Methods:** Eighty-four female artistic gymnasts, thirty-nine international and national level gymnasts [high-level; 14 [14, 15] y] and forty-five recreational and club level gymnasts [low-level; 14 [13, 15] y] completed the Eating Disorder Examination Questionnaire [EDE-Q 6.0], the RED-S knowledge Questionnaire and provided training details. **Results:** Seventeen gymnasts (20.2%) scored above the cutoff point on the EDE-Q. In addition, high-level gymnasts scored higher than low-level on EDE-Q [2.21 ± 1.37 (35.9%) vs. 1.19 ± 0.79 (6.7%), respectively; *p* < 0.001] and on its subscales: *Restraint, Eating Concerns, Weight Concerns,* and *Shape Concerns* [*p* < 0.001 to 0.009], thus indicating more severe disordered eating symptoms. No group differences were found in binge eating and compensatory behaviors. An important percentage of gymnasts reported at least one episode of binge eating and excessive training [39.3–58.3%], while four gymnasts reported self-induced vomiting. RED-S knowledge did not differ between groups. On average, gymnasts were unaware of correct answers related to RED-S [51.5%], its definitions [79.8–92.9%], and its association with menstrual disturbances and bone health [54.8–86.9%]. However, gymnasts reported better awareness of the impact of food restriction on illness and performance [47.6–84.5%]. **Conclusions:** Elite artistic gymnasts exhibited a higher prevalence of eating disorder symptoms than lower-level peers. Gymnasts at all levels demonstrated limited knowledge of the effects of RED-S on menstrual and bone health. Failure to recognize these risks may influence gymnasts’ eating behaviors and delay RED-S detection and management.

## 1. Introduction

Eating disorders are complex psychiatric disorders that may negatively affect health, development, and daily life [1]. They are often accompanied by other mental health disorders, their exact causes remain unclear, and their treatment is challenging, costly, and frequently ineffective [2]. Eating behaviors exist along a continuum, ranging from optimal nutritional practices to clinically diagnosable eating disorders, with disordered eating representing a midpoint [1]. Anorexia Nervosa and Bulimia Nervosa represent the most severe forms of disordered eating [3]. Subclinical behaviors—such as body image preoccupation, food restriction, bingeing, purging, and laxative or diuretic misuse—can negatively impact health and increase the risk of developing clinical eating disorders [3]. Intensive food restriction, and, as a result, low energy availability are the primary causes of Relative Energy Deficiency Syndrome (RED-S), a clinical syndrome characterized by an energy imbalance in which an athlete’s energy intake is insufficient to meet the demands of exercise, growth, and normal physiological functioning [4,5].

Previous studies have shown that participation in sports with strict demands on body weight, such as gymnastics, is associated with an increased risk of eating disorders in female athletes [6,7,8,9]. Success in these sports often necessitates maintaining a lean, sometimes prepubescent body, to meet sport-specific aesthetic demands and optimize performance [6,7,10,11]. Furthermore, high-level athletes frequently exhibit elevated rates of disordered eating compared to their lower-level or non-athlete counterparts, driven by performance pressures, appearance demands, and specific personality traits [12,13,14,15].

Eating disorders predominantly affect female adolescents and tend to follow a chronic course with significant impairment in social functioning [16]. Early diagnosis and timely therapeutic intervention are essential for improving long-term outcomes [17]. Adolescent athletes in aesthetic sports, such as artistic and rhythmic gymnastics, are often exposed to excessive training and competition loads during a critical developmental stage [18,19], when performance pressures coincide with significant physical changes [20,21]. The drive for a lean physique may lead to food restriction, and initial performance gains can reinforce this behavior, increasing the risk of eating disorders [22,23].

Importantly, a lack of understanding of the nutritional requirements of sport may influence athletes’ dietary habits and increase the risks of eating disorders and RED-S [4]. Research on RED-S has typically been performed among adolescent female athletes; however, emerging research demonstrates that male athletes are also affected, exhibiting clinical indicators of compromised bone health [24]. RED-S can result in a variety of adverse outcomes, including disruptions in hormonal regulation, growth retardation, impaired bone health, decreased immune function, and alterations in metabolic processes [25,26]. To lower the risk of RED-S, athletes, their families, and their support teams must be aware of its signs, symptoms, and consequences on sports performance, injury risk, recovery, and overall health. Failure to recognize these risks may not only influence gymnasts’ eating behavior but also delay early RED-S detection and management [27,28]. However, despite its significance, evidence on athletes’ awareness of the impact of RED-S on health and performance remains scarce. Thus, the aim of this study was to examine eating disorder symptoms and RED-S knowledge in a population of adolescent artistic gymnasts. It was hypothesized that: (a) a higher percentage of disturbed eating attitudes and behaviors would be observed in high-compared to low-level artistic gymnasts, and (b) a high percentage of gymnasts would be unaware of RED-S.

## 2. Materials and Methods

### 2.1. Participants

Eighty-four female artistic gymnasts, aged 12–18 years, participated in this research. Male athletes were excluded from the study due to an insufficient sample size, which would have limited the statistical power, prevented meaningful sex-based comparisons, and minimized confounding factors associated with sex-based physiological, behavioral, and nutritional differences.

The study questionnaire consisted of two parts: (a) a questionnaire which included age, training and competition details such as training experience (i.e., the years of systematic training and competing in artistic gymnastics), number of training sessions/hours per week and number of competitions per year, and (b) the Eating Disorder Examination Questionnaire (EDE-Q) [29,30], and the Knowledge of Relative Energy Deficiency Syndrome Questionnaire (RED-S Knowledge) [31]. The questionnaires were distributed to the participants in their training facilities, one hour before training. Athletes were gathered in small groups in a separate room, with no coaches or parents present. A member of the research team was present during questionnaire completion to provide clarification if needed and to ensure that participants remained focused and completed the inventories independently. Furthermore, athletes were informed about the confidentiality and anonymity of their responses and their right to withdraw from the study at any time without being required to provide any explanation for their decision. Participants responded to the questionnaire on paper, and a researcher was available to answer questions and collect the completed questionnaires.

The eighty-four artistic gymnasts included a population of international and national level gymnasts (High-level gymnasts; n = 39) and club and recreational level gymnasts (Low-level gymnasts; n = 45). The sample of high-level gymnasts (n = 39) comprised all the national team members of the country, trained six times per week, for approximately 3–4.5 h per session, and participated in competitions 3–4 times a year. Low-level artistic gymnasts trained 2–4 times per week, for 1.5–2.5 h per session, and participated in contests, festivals, or club competitions 2–3 times a year. The high-level artistic gymnasts were recruited in collaboration with the Hellenic Gymnastics Federation. The club and recreational level artistic gymnasts were randomly recruited from three different gymnastics clubs.

Participants’ Body Mass Index (BMI) was calculated as the ratio of body weight to the squared standing height (kg/m^2^). The characteristics of the participants are shown in Table 1. Prior to the study, the athletes and their parents were fully informed about the purpose and procedures of this study and gave written informed consent. Instructions to the participants included a reminder to respond to all items and a statement that there were no right or wrong answers. Procedures were approved by the local Institutional Ethics Review Committee (number 1645/15-05-2024) and followed the Code of Ethics set by the World Medical Association.

### 2.2. Study Measures

#### 2.2.1. Eating Disorder Examination Questionnaire (EDE-Q)

The Eating Disorder Examination Questionnaire (EDE-Q) [29,30] is a 28-item self-report questionnaire designed to assess the range and severity of features associated with a diagnosis of an eating disorder and is derived from the Eating Disorder Examination interview [32]. The EDE-Q focuses on the past 28 days and is scored using a 7-point, forced-choice rating scheme. Subscale scores, relating to *Dietary Restraint*, *Eating Concerns*, *Concerns about Shape*, and *Concerns about Weight*, and a global score, are derived from the 22 items addressing attitudinal aspects of eating-disorder psychopathology. The total score of the inventory is an average of the subscales. A global EDE-Q score of 2.612 for the Greek population yields an optimal trade-off to determine eating disorder diagnosis [30]. Questions 13 to 18 (6 items) of EDE-Q are individually examined to ascertain the frequency of binge eating and compensatory behaviors occurring during the past four weeks (28 days) [29,33]. These items do not contribute to the subscale scores. The questionnaire also includes three more questions, which are not numbered. The two questions ask the respondent to estimate his/her weight and height. The third question concerns the frequency of menstruation for the last 3–4 months, as well as the use of contraceptive pills. Cronbach’s α values for the total score of the questionnaire as well as its subscales were 0.840, 0.808, 0.817, 0.880, and 0.811, for EDE-Q total score, *Dietary Restraint*, *Eating Concerns*, *Concerns about Shape*, and *Concerns about Weight*, respectively.

#### 2.2.2. Knowledge of Relative Energy Deficiency Syndrome

This comprehensive 18-item questionnaire assesses theoretical and practical understanding of the signs, symptoms, and consequences of RED-S, including menstrual irregularities, poor bone health, impaired immunity, and decreased neuromuscular performance [31]. The domains of the inventory that assessed RED-S knowledge are labelled as follows: (1) Awareness and definition of Low Energy Availability (LEA), Athletic Triad, and RED-S (items 1–3); (2) knowledge of RED-S signs and symptoms (items 4–8); and (3) knowledge of health and performance consequences of RED-S (items 9–18). The questionnaire has high content as well as construct validity [31]. Total scores are calculated by summing the scores of 18-items. Preliminary analyses of the RED-S knowledge questionnaire’s reliability in the Greek population indicate acceptable reliability, with Cronbach’s alpha values around 0.70.

### 2.3. Statistical Analysis

The normality of distribution of continuous variables was assessed using the Shapiro–Wilks test and Q-Q plots. For normally distributed data, data are presented as means ± SD, and differences between high- and low-level artistic gymnasts were determined using independent samples *t*-tests. Data that were not normally distributed are presented as medians (Q1, Q3), while data on categorical variables are presented as frequencies (n, %); differences between high- and low-level artistic gymnasts were determined using Mann–Whitney rank or Chi-Square tests, respectively. Statistical significance was set at the 5% level (*p*-value < 0.05). All hypotheses tested were two-tailed. All analyses were performed using SPSS (version 29, SPSS Inc. Chicago, IL, USA).

## 3. Results

### 3.1. Eating Disorder Examination Questionnaire 

A total of 84 artistic gymnasts provided data for the variables of interest and were included in the analyses for the present study. High-level artistic gymnasts had more training experience, lower body mass, and BMI than low-level artistic gymnasts (*p* < 0.05, Table 1).

High-level artistic gymnasts scored higher than low-level in EDE-Q global score and its subscales Restraint, Eating Concerns, Weight Concerns, and Shape Concerns (*p* < 0.05; Table 2).

The six items of EDE were transformed into dichotomous variables, with 0 indicating the absence of binge eating and compensatory behaviors, and 1 indicating the presence of at least one episode. No differences were found between groups across these six EDE-Q items (*p* > 0.05). In contrast, more high-level artistic gymnasts had missed menstrual cycles in the last 3–4 months (*p* < 0.001), while no differences were observed between groups in contraceptive use (*p* = 0.283). Furthermore, when frequencies were computed for the six EDE-Q items for all the artistic gymnasts combined, it was found that 49 girls (58.3%) were concerned about the amount of food (EDE13) and 48 girls (57.2%) lost control of their eating at least once (EDE14). Forty-two (50%) experienced at least one subjective or objective binge-eating episode (EDE15). Four athletes (4.8%) reported self-induced vomiting as compensatory behavior after binge eating (EDE16). No gymnast reported laxative or diuretic misuse (EDE 17). Thirty-three athletes (39.3%) reported additional excessive training as compensatory behavior to avoid feeling guilty about eating (EDE18).

The percentage of artistic gymnasts scoring higher than the cut-off of 2.612 in EDE-Q was 20.2% (n = 17; 35.9% in high and 6.7% in low-level gymnasts). Those gymnasts scored significantly higher in all EDE-Q subscales (*p* < 0.001), with no significant differences in main characteristics (age, height, weight, BMI, training experience and RED-S knowledge score (*p* > 0.05); however, they were more likely to train at high than low level, compared to those scoring lower than 2.612 (*p* < 0.001).

### 3.2. RelativeED-S Knowledge

**Figure 1:**
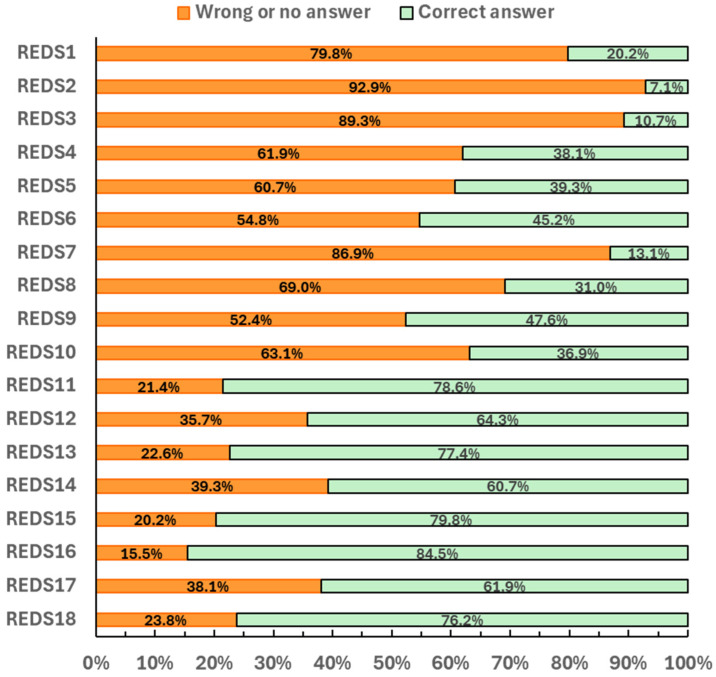
Percentage of correct and incorrect responses to the RED-S knowledge questionnaire among gymnasts (n = 84).

No differences were observed between groups in the total scores of the RED-S knowledge questionnaire (*p* = 0.961) (Table 2). Athletes’ responses to each question in this questionnaire are presented in Figure 1 and in Supplementary Table S1. In the domain assessing awareness and definition of LEA, Triad, and RED-S, 79.8 to 92.9% of the participants reported unawareness of these definitions. In the domain assessing knowledge of RED-S signs and symptoms, 54.8 to 86.9% of all artistic gymnasts failed to recognize known RED-S signs and symptoms and in the domain knowledge of the health and performance consequences of RED-S, 15.5 to 52.4% of the participants exhibited limited understanding of the impact of food restriction on their health and performance (Figure 1 and Supplementary Table S1). On average, 51.5% of artistic gymnasts provided incorrect answers related to RED-S.

## 4. Discussion

This study examined eating disorder symptoms and RED-S knowledge in adolescent artistic gymnasts. A higher percentage of eating disorder symptoms and higher total scores in EDE-Q and its subscales were found in high-level compared to lower-level artistic gymnasts. An important percentage of artistic gymnasts reported at least one episode of binge eating and excessive training. RED-S knowledge did not differ between groups, with most artistic gymnasts unaware of the main aspects of Relative Energy Deficiency in Sports.

It is well-established that eating disorders have a greater prevalence among high-performance athletes as compared with non-elite athletes and the general population, especially in disciplines that emphasize leanness, like gymnastics [10,22,34]. In a previous study with adolescent artistic gymnasts, Tan et al. [22] also reported that disordered eating among elite female artistic gymnasts was higher than that observed in lower-level gymnasts, and the same was found for international-level adolescent rhythmic gymnasts [35]. The finding that a large percentage (35.9%) of high-level adolescent artistic gymnasts in this study demonstrated eating disorder symptoms-based on their EDE-Q scores, is in line with previous research highlighting the need for psychological support and treatment in high-level sport environments [6,12]. In contrast, 6.7% of club and recreational artistic gymnasts scored higher than 2.612 in EDE-Q, a percentage almost similar to that found in Greek adolescents (9.8%) [36]. This shows that moderate exercise and lower performance and appearance demands are linked to reduced occurrence of disturbed eating attitudes compared to high-level competitive athletes (35.9% in the present study), while remaining comparable to the general adolescent population. High-level artistic gymnasts also scored higher than low-level in the subscales *Restraint*, *Eating Concerns*, *Weight Concerns*, and *Shape Concerns*. Artistic gymnasts are required to execute complex technical skills during flight while maintaining difficult body shapes. Thus, the ratio of force and power generation to body mass is a critical performance determinant in gymnastics from an early age, potentially leading youth gymnasts to engage in food restriction [37,38]. Notably, despite having a lower BMI and more extensive training experience, high-level artistic gymnasts in this study demonstrated greater concerns regarding weight and body shape compared to lower-level peers. Heightened preoccupation with weight and a drive for thinness are significant risk factors for the development of eating disorders [37,39,40].

No differences were found between groups regarding binge eating and compensatory behaviors. Over 50% of artistic gymnasts reported overeating with a loss of control and binge eating at least once in the past 28 days, highlighting the potential influence of the gymnastics environment (e.g., negative comments, performance anxiety) in triggering binge eating as a coping mechanism for emotional distress, guilt, or shame [39,41]. Regarding compensatory behaviors, fewer gymnasts reported excessive training or self-induced vomiting, with approximately 4% engaging in self-induced vomiting. Similar percentages of self-vomiting, a symptom associated with severe health, psychological, and, in some cases, life-threatening consequences, have also been reported in previous studies in elite athletes [42,43]. While the athletes’ responses in these areas are concerning, these findings must be interpreted with caution. Previous research involving Greek adolescents has highlighted discrepancies between the six items of the EDE-Q and objectively assessed compensatory behaviors during clinical evaluations [30]. Similarly, Mond et al. [44] found that about 42% of the participants who reported self-induced vomiting or laxative misuse on a questionnaire denied these behaviors when they were questioned in a face-to-face interview. Thus, these findings highlight the need to interpret results from self-report inventories, such as the EDE-Q, within the framework of clinical evaluation. While inventories offer practical benefits, including ease of use and preliminary insights, accurate diagnosis of eating disorders requires validation through clinical interviews and comprehensive diagnostic assessments.

RED-S is common among female athletes at different ages and performance levels and results in serious health and performance consequences [45,46,47]. Exposure to low energy availability is the main underlying etiological factor thus, awareness of the effects of food restriction on metabolic rate, menstrual, bone, cardiovascular, and gastroenterological health, immunity, and overall well-being is of paramount importance and especially for adolescent athletes [48]. The results of this study highlight an interesting finding: irrespective of performance level, most artistic gymnasts reported unawareness of the RED-S definition, and over 50% of them failed to recognize known associations between food restriction and menstrual and bone health. This lack of awareness was not specific to high or low performance levels but rather reflects a broader, systemic gap in knowledge among athletes, and possibly coaches, or parents. Limited understanding of RED-S and its health implications may delay recognition and treatment of serious conditions, placing athletes at risk [49].

The finding that 86.9% of gymnasts responded “No or I don’t know” to the question about whether amenorrhea increases the risk of fractures highlights a substantial educational gap among athletes, coaches, and parents. This lack of awareness is especially concerning given the well-established association between menstrual dysfunction and impaired bone health, which significantly increases the risk of stress fractures. Ackerman et al. [50] reported that adolescent athletes with amenorrhea had a markedly higher lifetime incidence of fractures (47%) compared to their eumenorrheic peers (25.7%) and non-athletes (12.5%). In artistic gymnastics, the prevalence of stress fractures is further amplified by the sport’s high mechanical loads imposed on skeletally immature athletes. A recent systematic review found that women’s gymnastics has one of the highest rates of stress fractures among collegiate sports, with an incidence of 25.58 per 100,000 athlete-exposures [51]. In line with this finding, a previous survey of 712 adolescent and young adult runners, dancers, and figure skaters found that only 12% had heard of the female athlete triad and only 7% were able to name two of the three components of the triad [52]. Anecdotally, over 60% of the artistic gymnasts in this study reported that it is normal for female athletes to miss their period when they are following intensive training regimes. This result aligns with the finding that high-level artistic gymnasts reported a greater number of missed menstrual cycles compared to low-level peers over the past 3–4 months. At the elite level, misconceptions regarding menstruation are prevalent, with evidence suggesting that 28–56% of female adolescent athletes believe that the absence of menstruation is a normal consequence of intense athletic training [53,54]. Several studies on female athletes highlight a culture that encourages the pursuit of the ideal body through restrictive eating and excessive training, cultivating a “lighter is better” mentality from the early stages of their careers, while often neglecting the significance of menstrual disturbances [54,55]. Nevertheless, artistic gymnasts in this study demonstrated a better understanding of the effects of severe food restriction on immunity and neuromuscular performance, likely influenced by feedback from coaches and parents or their own personal experiences. It is plausible that malnourished athletes face performance difficulties due to weakness and energy depletion.

Although this study is the first to examine awareness of energy deficiency and eating disorder symptoms in adolescent artistic gymnasts, there are some limitations that should be acknowledged. EDE-Q, when using its global score, is a proper screening tool for assessing the core psychopathology of eating disorders [30,33]. However, the assessment of pathological behaviors using the six items of the EDE-Q has limitations within the Greek population, as adolescents may misinterpret terms such as “large amount of food” and “loss of control” or misunderstand the motivation behind excessive exercise [30,33]. They may perceive the term as referring to additional exercise for physical fitness rather than compensatory behavior. Therefore, it is crucial to recognize that the results obtained from these self-report questions should be cross-validated with findings from face-to-face interviews. A second limitation is that this study relied solely on self-report instruments. Thus, a comprehensive clinical evaluation is necessary to accurately assess the presence of eating disorders. In addition, data on menstruation were derived solely from self-reported responses to non-standardized items included in the EDE-Q. These items, which asked about menstrual frequency and contraceptive use over the past 3–4 months, may be subject to recall bias or misreporting, potentially affecting the accuracy and reliability of the menstrual data collected. A final limitation is the small sample size of high-level adolescent artistic gymnasts and unequal recruitment methods, which restrict the statistical power to detect small or medium effect sizes and may introduce bias. However, it should be reported that the sample of high-level gymnasts (n = 39) comprised all female artistic gymnasts in Greece who were members of the national team and trained at the two high-performance centers located in Athens and Thessaloniki. For comparison purposes, a demographically similar group of club and recreational-level artistic gymnasts (n = 45) was randomly selected from three different gymnastics clubs. The inclusion of a large sample of elite athletes was crucial for investigating sensitive issues like eating disorders and RED-S awareness.

Nevertheless, the findings of this study highlight the need for the gymnastics community to educate athletes and coaches on the importance of maintaining adequate energy availability to optimize both health and performance. It is crucial to teach athletes effective fueling strategies for different training durations and intensities, as well as for growth [26]. Adolescent aesthetic athletes who fail to recognize potential risks may gradually develop pathological behaviors, eventually losing control over their actions, which can compromise both their health and performance. The treatment of RED-S and low energy availability necessitates addressing the underlying causes [25]. Researchers and specialists should focus on strengthening protective factors (e.g., enhancing self-esteem and a positive body image, encouraging acceptance of physical changes during adolescence, and improving media literacy) while reducing risk factors (e.g., internalization of the stereotype of the “ideal body”, peer pressure, and fat shaming), involving the entire athlete support system [25,26]. In addition, long-term coach education programs, especially those offered at the university level or through national sport federations, should include modules on RED-S and training load management. To further promote awareness, workshops and digital resources should also be made available to parents and athletes through National Olympic Committees and sport federations. Increasing knowledge in these areas can help identify additional educational needs and support athletes, coaches, and sports medicine professionals in making informed decisions about training, competition, and recovery. Failure to recognize these risks may not only impact artistic gymnasts’ eating behaviors but also hinder the early detection and management of RED-S, where timely intervention may be required.

## 5. Conclusions

A higher prevalence of eating disorder symptoms was observed among high-level artistic gymnasts compared to their lower-level counterparts, suggesting that reduced performance and appearance-related pressures may be associated with fewer disordered eating behaviors. The finding that RED-S knowledge did not differ between groups—and that most artistic gymnasts were unaware of the core aspects of Relative Energy Deficiency in Sport—is both novel and concerning. This widespread lack of awareness was not limited to athletes of a specific performance level, but instead highlights a broader, systemic gap in understanding that may extend to coaches and parents as well. 

## Figures and Tables

**Table 1:** Age, competitive experience, and anthropometric characteristics of the participants ^1^.

	High Level Gymnasts (n = 39)	Low Level Gymnasts (n = 45)	*p*-Value
Age (y)	14 (14, 15)	14 (13, 15)	0.061
Training experience (y)	8 (6, 8)	6 (3, 8)	<0.001
Height (cm)	159 (157, 162)	160 (156, 163)	0.815
Body mass (Kg)	47 (44, 50)	50 (47, 55)	0.019
Body Mass Index (kg/m^2^)	18.7 (17.7, 19.7)	20.1 (18.4, 21.4)	0.006

^1^ Note: Values are expressed as median (Q1, Q3). *p*-values derived from Mann–Whitney rank test. Statistical significance was set at the 5% level.

**Table 2:** Participants’ values of the EDE-6 Questionnaire ^1^.

	High Level Gymnasts (n = 39)	Low Level Gymnasts (n = 45)	*p*-Value
EDE-Q total score	2.07 (1.12, 3.13)	1.17 (0.44, 1.73)	<0.001
Subscales			
*Restrain*	1.40 (0.60, 3.60)	0.60 (0.20, 1.70)	0.014
*Eating Concern*	1.20 (0.40, 2.60)	0.60 (0.20, 1.20)	0.009
*Weight Concern*	2.00 (1.60, 4.20)	1.00 (0.40, 2.20)	<0.001
*Shape concern*	2.38 (1.63, 3.75)	1.25 (0.50, 2.31)	<0.001
RED-S total score	8.74 ± 3.04	8.71 ± 2.98	0.961

^1^ Note: Values are expressed as median (Q1, Q3) for EDE-Q and its subscales and as means ± SD for REDS knowledge score. *p*-values derived from Mann–Whitney rank test and from *t*-test. Statistical significance was set at the 5% level.

## Data Availability

All data will be made available on request to the corresponding author.

## Supplementary Materials

The following supporting information can be downloaded at: https://www.mdpi.com/article/10.3390/nu17101699/s1. Table S1: Gymnasts’ responses to the RED-S knowledge questionnaire (n = 84).

## Abbreviations

The following abbreviations are used in this manuscript:

RED-S	Relative Energy Deficiency Syndrome
EDE-Q	Eating Disorder Examination Questionnaire
LEA	Low energy availability

## References

[1] American Psychiatric Association (2013). Diagnostic and Statistical Manual of Mental Disorders.

[2] Fisher M. (2006). Treatment of eating disorders in children, adolescents, and young adults. Pediatr. Rev..

[3] Garner D., Fairburn C.G., Brownell K.D. (2002). Measurement of eating disorder psychopathology. Eating Disorders and Obesity: A Comprehensive Handbook.

[4] Mountjoy M., Ackerman K.E., Bailey D.M., Burke L.M., Constantini N., Hackney A.C., Heikura I.A., Melin A., Pensgaard A.M., Stellingwerff T. (2023). International Olympic Committee’s (IOC) consensus statement on relative energy deficiency in sport (REDs). Br. J. Sports Med..

[5] Angelidi A.M., Stefanakis K., Chou S.H., Valenzuela-Vallejo L., Dipla K., Boutari C., Ntoskas K., Tokmakidis P., Kokkinos A., Goulis D.G. (2024). Relative energy deficiency in sport (REDs): Endocrine manifestations, pathophysiology and treatments. Endocr. Rev..

[6] Sundgot-Borgen J. (1996). Eating disorders, energy intake, training volume, and menstrual function in high-level modern rhythmic gymnasts. Int. J. Sport Nutr..

[7] Okano G., Holmes R.A., Mu Z., Yang P., Lin Z., Nakai Y. (2005). Disordered eating in Japanese and Chinese female runners, rhythmic gymnasts, and gymnasts. Int. J. Sports Med..

[8] Anderson C., Petrie T.A. (2012). Prevalence of disordered eating and pathogenic weight control behaviors among NCAA Division I female collegiate gymnasts and swimmers. Res. Q. Exerc. Sport.

[9] de Bruin A.P., Oudejans R.R.D., Bakker F.C. (2007). Dieting and body image in aesthetic sports: A comparison of Dutch female gymnasts and non-aesthetic sport participants. Psychol. Sport Exerc..

[10] Van Durme K., Goossens L., Braet C. (2012). Adolescent aesthetic athletes: A group at risk for eating pathology?. Eat. Behav..

[11] Kontele I., Vassilakou T., Donti O. (2022). Weight pressures and eating disorder symptoms among adolescent female gymnasts of different performance levels in Greece. Children.

[12] Reardon C.L., Hainline B., Aron C.M., Baron D., Baum A.L., Bindra A., Budgett R., Campriani N., Castaldelli-Maia J.M., Currie A. (2019). Mental health in elite athletes: International Olympic Committee consensus statement. Br. J. Sports Med..

[13] Byrne S., McLean N. (2002). Elite athletes: Effects of the pressure to be thin. J. Sci. Med. Sport.

[14] Sundgot-Borgen J., Torstveit M.K. (2004). Prevalence of eating disorders in elite athletes is higher than in the general population. Clin. J. Sport Med..

[15] Coelho G.M., Soares Ede A., Ribeiro B.G. (2010). Are female athletes at increased risk for disordered eating and its complications?. Appetite.

[16] Golden N.H., Schneider M., Wood C. (2016). Preventing obesity and eating disorders in adolescents. Pediatrics.

[17] Ward Z.J., Rodriguez P., Wright D.R., Austin S.B., Long M.W. (2019). Estimation of eating disorders prevalence by age and associations with mortality in a simulated nationally representative US cohort. JAMA Netw. Open.

[18] Malina R.M., Baxter-Jones A.D.G., Armstrong N., Beunen G.P., Caine D., Daly R.M., Lewis R.D., Rogol A.D., Russell K. (2013). Role of intensive training in the growth and maturation of artistic gymnasts. Sports Med..

[19] Donti O., Bogdanis G.C., Kritikou M., Donti A., Theodorakou K. (2016). The relative contribution of physical fitness to the technical execution score in youth rhythmic gymnastics. J. Hum. Kinet..

[20] Desbrow B., McCormack J., Burke L.M., Cox G.R., Fallon K., Hislop M., Logan R., Marino N., Sawyer S.M., Shaw G. (2014). Sports Dietitians Australia position statement: Sports nutrition for the adolescent athlete. Int. J. Sport Nutr. Exerc. Metab..

[21] Bingham M.E., Borkan M., Quatromoni P. (2015). Sports nutrition advice for adolescent athletes: A time to focus on food. Am. J. Lifestyle Med..

[22] Tan J.O.A., Calitri R., Bloodworth A., McNamee M.J. (2016). Understanding eating disorders in elite gymnastics: Ethical and conceptual challenges. Clin. Sports Med..

[23] Jagim A.R., Fields J., Magee M.K., Kerksick C.M., Jones M.T. (2022). Contributing factors to low energy availability in female athletes: A narrative review of energy availability, training demands, nutrition barriers, body image, and disordered eating. Nutrients.

[24] Stenqvist T.B., Melin A.K., Torstveit M.K. (2023). Relative Energy Deficiency in Sport (REDs) indicators in male adolescent endurance athletes: A 3-year longitudinal study. Nutrients.

[25] Torstveit M.K., Ackerman K.E., Constantini N., Holtzman B., Koehler K., Mountjoy M.L., Sundgot-Borgen J., Melin A. (2023). Primary, secondary and tertiary prevention of Relative Energy Deficiency in Sport (REDs): A narrative review by a subgroup of the IOC consensus on REDs. Br. J. Sports Med..

[26] Torstveit M.K. (2004). The Female Athlete Triad in Norwegian Elite Athletes and Non-Athletic Controls: Identification and Prevalence of Disordered Eating, Menstrual Dysfunction, and Osteoporosis. Doctoral Dissertation.

[27] Burke L.M., Lundy B., Fahrenholtz I.L., Melin A.K. (2018). Pitfalls of conducting and interpreting estimates of energy availability in free-living athletes. Int. J. Sport Nutr. Exerc. Metab..

[28] Fostervold-Mathisen T., Ackland T., Burke L.M., Constantini N., Haudum J., Macnaughton L.S., Meyer N.L., Mountjoy M., Slater G., Sungot-Borgen J. (2023). Best practice recommendations for body composition considerations in sport to reduce health and performance risks: A critical review, original survey and expert opinion by a subgroup of the IOC consensus on Relative Energy Deficiency in Sport (REDs). Br. J. Sports Med..

[29] Fairburn C.G., Beglin S., Fairburn C.G. (2008). Eating Disorder Examination Questionnaire (EDE-Q 6.0). Cognitive Behavior Therapy and Eating Disorders.

[30] Pliatskidou S., Samakouri M., Kalamara E., Papageorgiou E., Koutrouvi K., Goulemtzakis C., Nikolaou E., Livaditis M. (2015). Validity of the Greek Eating Disorder Examination Questionnaire 6.0 (EDE-Q-6.0) among Greek adolescents. Psychiatriki.

[31] Pai N.N., Brown R.C., Black K.E. (2022). The development and validation of a questionnaire to assess relative energy deficiency in sport (RED-S) knowledge. J. Sci. Med. Sport.

[32] Fairburn C.G., Cooper P.J., Fairburn C.G., Wilson G.T. (1993). The Eating Disorder Examination. Binge Eating: Nature, Assessment And treatment.

[33] Pliatskidou S., Samakouri M., Kalamara E., Goulemtsakis C., Koutrouvi K., Papageorgiou E., Livadites M. (2012). Reliability of the Greek version of the Eating Disorder Examination Questionnaire (EDE-Q) in a sample of adolescent students. Psychiatriki.

[34] Bratland-Sanda S., Sundgot-Borgen J. (2013). Eating disorders in athletes: Overview of prevalence, risk factors, and recommendations for prevention and treatment. Eur. J. Sport Sci..

[35] Donti O., Donti A., Gaspari V., Pleksida P., Psychountaki M. (2021). Are they too perfect to eat healthy? Association between eating disorder symptoms and perfectionism in adolescent rhythmic gymnasts. Eat. Behav..

[36] Kyrgios I., Papageorgiou V., Kotanidou E., Kokka P., Kleisarchaki A., Mouzaki K., Tsara I., Efstratiou E., Haidich A.-B., Galli-Tsinopoulou A. Eating Disorders in Greek Adolescents: Frequency and Characteristics. Proceedings of the 54th Annual ESPE.

[37] Bacciotti S., Baxter-Jones A., Gaya A., Maia J. (2017). The physique of elite female artistic gymnasts: A systematic review. J. Hum. Kinet..

[38] Claessens A.L., Lefevre J., Beunen G., Malina R.M. (1999). The contribution of anthropometric characteristics to performance scores in elite female gymnasts. J. Sports Med. Phys. Fit..

[39] de Bruin A.P.K., Oudejans R.R.D., Bakker F.C., Woertman L. (2011). Contextual body image and athletes’ disordered eating: The contribution of athletic body image to disordered eating in high-performance women athletes. Eur. Eat. Disord. Rev..

[40] Donti O., Theodorakou K., Kambiotis S., Donti A. (2012). Self-esteem and trait anxiety in girls practicing competitive and recreational gymnastics. Sci. Gymnast. J..

[41] Kristjánsdóttir H., Sigurðardóttir P., Jónsdóttir S., Þorsteinsdóttir G., Saavedra J. (2019). Body image concern and eating disorder symptoms among elite Icelandic athletes. Int. J. Environ. Res. Public Health.

[42] Killen J.D., Taylor C.B., Telch M.J., Saylor K.E., Maron D.J., Robinson T.N. (1986). Self-induced vomiting and laxative and diuretic use among teenagers. J. Am. Med. Assoc..

[43] Rosen L.W., McKeag D.B., Hough D.O., Curley V. (1986). Pathogenic weight-control behavior in female athletes. Physician Sportsmed..

[44] Mond J.M., Hay P.J., Rodgers B., Owen C., Mitchell J.E. (2006). Correlates of self-induced vomiting and laxative misuse in a community sample of women. J. Nerv. Ment. Dis..

[45] Wasserfurth P., Palmowski J., Hahn A., Krüger K. (2020). Reasons for and consequences of low energy availability in female and male athletes: Social environment, adaptations, and prevention. Sports Med. Open.

[46] Torstveit M.K., Fahrenholtz I.L., Lichtenstein M.B., Stenqvist T.B., Melin A.K. (2019). Exercise dependence, eating disorder symptoms, and biomarkers of relative energy deficiency in sports (RED-S) among male endurance athletes. BMJ Open Sport Exerc. Med..

[47] Villa M., Villa-Vicente J.G., Seco-Calvo J., Mielgo-Ayuso J., Collado P.S. (2021). Body composition, dietary intake, and the risk of low energy availability in elite-level competitive rhythmic gymnasts. Nutrients.

[48] Logue D.M., Madigan S.M., Melin A., Delahunt E., Heinen M., Donnell S.-J.M., Corish C.A. (2020). Low energy availability in athletes 2020: An updated narrative review of prevalence, risk, within-day energy balance, knowledge, and impact on sports performance. Nutrients.

[49] Mountjoy M., Sundgot-Borgen J., Burke L., Carter S., Constantini N., Lebrun C., Meyer N., Sherman R., Steffen K., Budgett R. (2014). The IOC consensus statement: Beyond the female athlete triad—Relative energy deficiency in sport (RED-S). Br. J. Sports Med..

[50] Ackerman K.E., Sokoloff N.C., Maffazioli G.D.N., Clarke H., Lee H., Misra M. (2015). Fractures in relation to menstrual status and bone parameters in young athletes. Med. Sci. Sports Exerc..

[51] Campbell R.A., Bradshaw E.J., Ball N.B., Pease D.L., Spratford W. (2019). Injury epidemiology and risk factors in competitive artistic gymnasts: A systematic review. Br. J. Sports Med..

[52] Tosi M., Maslyanskaya S., Dodson N.A., Coupey S.M. (2019). The female athlete triad: A comparison of knowledge and risk in adolescent and young adult figure skaters, dancers, and runners. J. Pediatr. Adolesc. Gynecol..

[53] Torstveit M.K., Sundgot-Borgen J. (2005). Participation in leanness sports but not training volume is associated with menstrual dysfunction: A national survey of 1276 elite athletes and controls. Br. J. Sports Med..

[54] Calcaterra V., Vandoni M., Bianchi A., Pirazzi A., Tiranini L., Baldassarre P., Diotti M., Cavallo C., Nappi R.E., Zuccotti G. (2024). Menstrual dysfunction in adolescent female athletes. Sports.

[55] Sherman R.T., Thompson R.A., Rose J.S. (1996). Body mass index and athletic performance in elite female gymnasts. J. Sport Behav..

