# Supplementary material for: Eating Disorder Symptoms and Energy Deficiency Awareness in Adolescent Artistic Gymnasts: Evidence of a Knowledge Gap
Source: Nutrients. 2025 May 16;17(10):1699. doi: 10.3390/nu17101699 (PMC12114068; doi:10.3390/nu17101699)
Supplement: Supplementary file 1 [file nutrients-17-01699-s001.zip › nutrients-3571187-supplementary.pdf]

Supplementary Table S1. Gymnasts’ responses to the RED-S knowledge questionnaire (n= 84)

Question	Response option	Number of gymnasts (%)
Have you ever heard of Low Energy Availability (LEA) ?	Yes	17 (20.2%)
	No	67 (79.8%)
Have you ever heard of Relative Energy Deficiency in Sports (RED-S)?	Yes	6 (7.1%)
	No	78 (92.9%)
Have you ever heard of Female Athlete Triad (Triad)?	Yes	9 (10.7%)
	No	75 (89.3%)
Having an irregular menstrual cycle is often a sign that female athletes/active individuals are in peak competitive shape	True/Unsure	52 (61.9%)
	False	32 (38.1%)
Do you think it is normal for female athletes/active individuals to miss their periods? (excluding pregnancy or purposely skipping periods by contraception)?	Yes/ Not sure/Depends on the situation	51 (60.7%)
	No	33 (39.3%)
Do you think that not consuming enough energy could result in the absence of periods	Yes	38 (45.2%)
	No/Not sure	46 (54.8%)
Do you think fractures (very small cracks or breaks) occur more often in girls/women who miss their period for 3 or more months than in girls/women who have regular periods? (excluding pregnancy or purposely skipping periods by contraception)	Yes	11 (13.1%)
	No/Not sure	73 (86.9%)
Do you think irregular or the absence of periods is associated with developing weaker bone?	Yes	26 (31%)
	No/Not sure	58 (69%)
Which of the following can increase an athlete's risk of infections such as cold and flu? (please tick all that apply)	Intensive training with inadequate rest	40 (47.6%)
	No/Don’t know	44 (52.4%)
	Insufficient energy intake	31 (36.9%)
	Insufficient intake of fluids’/ Don’t know	53 (63,1%)
Which of the following do you think could result from chronic insufficient energy? (please tick all that apply)	Treat as eleven different items – this means that those who don’t tick the false items, get a point for that too. 1= ticked; 0= non-ticked	
Increased sprint performance	Ticked	18 (21.4%)
	Non-ticked	66 (78.6%)
Decreased sprint performance	Non-ticked	30 (35.7%)
	Ticked	54 (64.3%)
Weight changes	Non-ticked	19 (22.6%)
	Ticked	65 (77.4%)
Body composition changes	Non-ticked	33 (39.3%)
	Ticked	51 (60.7%)
Decreased muscle strength	Non-ticked	17 (20.2%)
	Ticked	67 (79.8%)
Increased muscle strength	Ticked	13 (15.5%)
	Non-ticked	71 (84.5%)
Decreased muscle mass	Non-ticked	32 (38.1%)
	Ticked	52 (61.9%)
Increased injuries	Non-ticked	20 (23.8%)
	Ticked	64 (76.2%)
